# Illustrating User Needs for eHealth With Experience Map: Interview Study With Chronic Kidney Disease Patients

**DOI:** 10.2196/48221

**Published:** 2025-03-18

**Authors:** Paula Valkonen, Sini Hölsä, Johanna Viitanen, Sini Leinonen, Nina Karisalmi, Virpi Rauta

**Affiliations:** 1 Department of Computer Science Aalto University Espoo Finland; 2 Department of Nephrology University of Helsinki Helsinki University Central Hospital Helsinki Finland; 3 Helsinki University Hospital Abdominal Center Nephrology Helsinki Finland

**Keywords:** user need, chronic illness, kidney disease, older adult, eHealth, experience map, human-centered design, home dialysis

## Abstract

**Background:**

Chronic kidney disease (CKD) is a common condition worldwide and home dialysis (HD) provides economic, quality of life, and clinical advantages compared to other dialysis modalities. Human-centered design aims to support the development of eHealth solutions with high usability and user experience. However, research on the eHealth needs of patients using HD is scarce.

**Objective:**

This study aimed to support the design of eHealth for patients with CKD, particularly for patients using HD, by developing a kidney disease experience map that illustrates user needs, concerns, and barriers. The research questions were (1) what experiences do patients, particularly older adults, have in their everyday lives with CKD? (2) what user needs do patients with CKD have for HD eHealth? (3) how can these needs be illustrated using the experience map technique? The study focused on patients aged >60 years, as they are at a higher risk of chronic conditions. The study was conducted as part of the eHealth in HD project, coordinated by Hospital District of Helsinki and Uusimaa, Finland.

**Methods:**

In total, 18 patients in different care modalities participated in retrospective interviews conducted between October 2020 and April 2021. The interviews included a preliminary task with patient journey illustrations and questions about their experiences and everyday lives with CKD. The data analysis was conducted using a thematic analysis approach and the process included several phases.

**Results:**

On the basis of the thematic analysis, 5 categories were identified: healthy habits, concerns about and barriers to eHealth use, digital communication, patients’ emotions, and everyday life with CKD. These were illustrated in the first version of the kidney disease experience map. The patients had different healthy habits regarding social life, sports, and other activities. They had challenges with poorly functioning eHealth software and experienced other factors, such as a lack of interest and lack of skills for eHealth use. Technical devices do not always meet the emotional or physical needs of their users. This caused feelings of frustration, worry, and fear in patients, yet also fostered situational awareness and hope.

**Conclusions:**

The experience map is a promising method for illustrating user needs and communicating the patient’s voice for eHealth development. eHealth offers possibilities to support patient’s everyday life with chronic disease. The patient’s situation and capacity to use eHealth solutions vary with their everyday challenges, opportunities, and their current stage of treatment. The kidney disease experience map will be used and further developed in the ongoing research project “Better Health at Home—Optimized Human-Centered Care of Predialysis and Home Dialysis Patients” (2022 to 2026).

## Introduction

### Background

Chronic kidney disease (CKD) is a common condition worldwide. It has been estimated that 11% to 13% of the population in high-income countries have CKD. The number of patients with advanced kidney disease is growing 5% to 7% per year [1,2]. The prevalence of CKD is highest among older adults, ranging from 38% to 44% in patients aged >65 years [3,4]. In total 2.05 million people were treated with dialysis worldwide in 2010 [2]. In 2017, 1.2 million people died globally because of CKD [5].

Patients with end-stage CKD need kidney transplants or dialysis to survive. Dialysis can be performed in a dialysis unit in a hospital (ie, in-center dialysis), in a satellite dialysis unit, or at home using peritoneal dialysis or hemodialysis. The dialysis modality may vary depending on the patient’s current health and life situation. Both in-center and satellite dialysis can be laborious for patients [6] and impose a heavy financial burden on medical care [7]. Therefore, home dialysis (HD) provides better quality of life and clinical advantages and empowers patients by providing them with more flexibility in their everyday lives [6,8].

Even though bringing dialysis treatment to a patient’s home might be burdensome and complicated for both the patient and the health care unit [8,9], there is a common understanding that HD prevalence needs to increase [1]. HD creates opportunities to improve the patient’s safety and quality of life, as well as to support self-management of health [6,10]. HD has also proven to be feasible for older adults, even though challenges such as fear of needles or doubts about handling them, or other physical limitations have been recognized [8,11,12].

Research advises a holistic eHealth design that integrates technologies, end users, and use contexts [13-15]. Using human-centered design (HCD) approach [16], our study aimed to support the design of eHealth for patients with CKD, especially those on HD care, by investigating user needs, concerns, and digital service barriers, and illustrating those in the format of a kidney disease experience map. Due to the high prevalence of CKD among older adults [8], both in Finland and worldwide [17], the focus of the study was set on the perspective of older adults. The research questions were as follows:

1. What experiences do patients, particularly older adults, have in their everyday lives with CKD?
2. What user needs do patients with CKD have for HD eHealth?
3. How can these needs be illustrated using the experience map technique?

**Figure 1:**
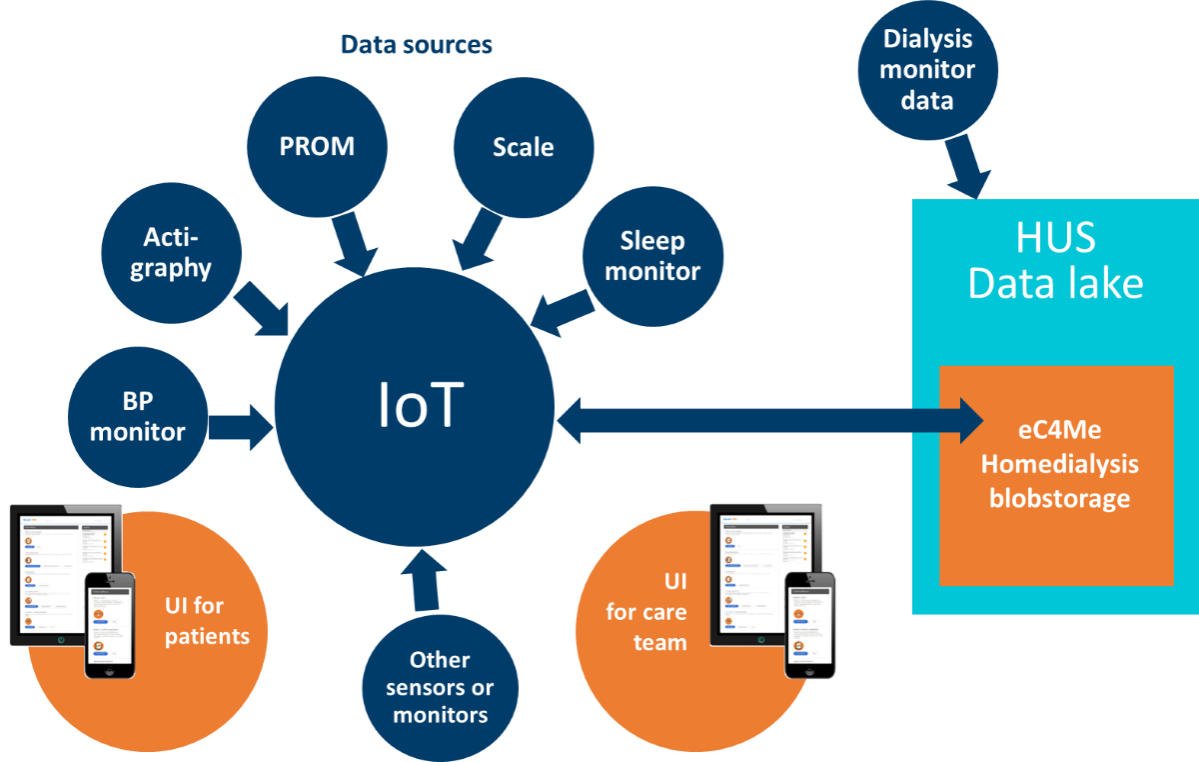
eHealth solution and research devices for home dialysis. BP: blood pressure; HUS: Helsinki University Hospital; IoT: Internet of Things; PROM: patient-reported outcome measure; UI: user interface.

The study was part of the eHealth in HD project (“device research”) [18] coordinated by the Hospital District of Helsinki and Uusimaa in Finland from 2020 to 2022. The project aimed to create a novel eHealth solution for patients with CKD undergoing HD (named “Home dialysis eHealth solution”) and research devices that support patients both before and throughout dialysis therapy by gathering monitoring data, managing HD supply orders, and facilitating communication between the patient and the health care team (Figure 1).

### HCD of eHealth Solutions

HCD is an approach to interactive system development that aims to develop solutions with high usability and user experience [16]. The principles of HCD highlight the importance of involving end users in an iterative design process [16,19] and understanding their needs at the beginning of the development process [16,19].

When exploring user needs, researchers collect information, for example, about what features digital services should contain and how they should function from the perspectives of end users. The user needs exploration is important as it helps service designers to understand the role and meaning of the solutions for end users, in our case, patients using HD. eHealth is used to refer to digital health services, and health information offered through the internet or other technology solutions improving health care [20,21]. By designing in a human-centric way and involving end users in the development, eHealth can offer more value and new ways to provide health care and well-being services for patients and all citizens [13,21]. For patients with chronic illnesses, eHealth solutions can offer new communication possibilities between the patient and the health care team, as well as support empowerment and improve quality of life [21,22].

### Patient Journey Maps and Experience Maps as Illustrations of User Needs

A patient journey map and an experience map are tools for 2 different purposes. In this study, patient journey maps were used as a part of patient interviews [23], and an experience map was used to communicate the results of the study holistically.

Patient journey maps as tools for visualizing patient journeys can be used to support the design of eHealth in several ways. The maps can help researchers and designers to identify how health care processes can be improved, for example, identify gaps and improve the processes by integrating eHealth solutions as part of those processes [24]. The patient journey maps sum up patients’ experiences and care activities in a single, chronological, timeline-type visualization [24]. As the maps can help to explain what patients go through with their disease [23,24], they can help designers promote empathy, which is important in HCD [25-27]. Furthermore, they can help the sharing of knowledge between stakeholders as well as activate creative thinking [28].

Patient journey maps can be applied in various specialties regardless of the medical condition. For example, patient journey maps have been used to illustrate home isolation experiences of people with mild COVID-19 [29], hypertensive disorders in pregnancy [30], cancer [24], cervical dystonia [31], and kidney diseases [32]. In literature, the focus of patient journey maps varies from communicating patients’ emotions and identifying process gaps to the importance of shared decision-making [33].

Experience maps are tools to combine situations, functions, emotions, and contacts in the same visualization [34]. Experience maps are valuable in the development of various eHealth, mobile health, and other apps, helping to communicate, for example, how cancer affects a patient’s life and capturing patients’ voices [35]. However, similar to patient journey maps, there is no standardized way to visualize and use them in an eHealth development context [33]. To our knowledge, no experience maps have been produced to represent the everyday life of patients with CKD using HD. Even though working with experience maps helps to identify users’ needs [34], based on our literature review, few patient journey maps or experience maps focus on illustrating and communicating the needs as a basis for eHealth design and requirements specification.

### Older Adults as eHealth Users

The European population is aging [36]. In Finland, >2 million citizens (ie, 36% of the population) are ≥55 years old [37]. Finnish older adults have good self-confidence in using digital services. In the 55 to 64 age group, >80% of Finns felt that their digital skills were at least on par with those of other Finns, and in the 65 to 74 age group, >60% of Finns felt the same [38]. At the same time, the number of patients in Finland who undergo dialysis, is expected to increase by >36% before 2040 [39]. Therefore, Finnish older adults seem to have both a need for and self-confidence in using CKD eHealth.

Older adults, due to an increase in diseases and complex health issues, could benefit substantially from eHealth [40]. Still, they experience several barriers to using eHealth, such as privacy concerns, lack of motivation to use digital solutions [41,42], and challenges to finding, accessing and understanding health-related information [40,43]. In addition, they have concerns about eHealth reducing the time with the physicians during appointments [42]. Although they can benefit from eHealth, they still face several challenges that could be solved with careful design.

The health situation also affects the use of the eHealth and user needs. Many eHealth tools are intended for the treatment of a specific disease. One or more chronic health conditions can lead to several regular self-management tasks [40], which can potentially affect a patient’s everyday life and the use context of eHealth. Therefore, eHealth should be adapted to each user’s special situation [44].

## Methods

### Overview

**Figure 2:**
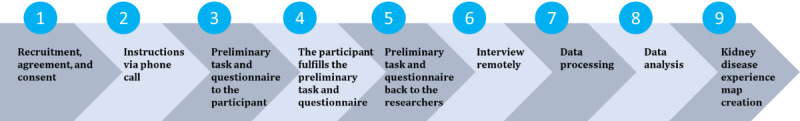
Procedure of the study.

Combining different context-specific strategies is important when developing services in a complex environment, such as health care [15]. Our study on user needs of patients with CKD for eHealth used a qualitative research approach and semistructured interviews [23,45] as the primary method for data gathering. The interviews were supported with preliminary tasks, which included visualizing patient’s journey in the form of a timeline drawing [23]. The procedure of the study included several phases starting from the recruitment and ending with the creation of the kidney disease experience map illustration (Figure 2).

### Ethical Considerations

The empirical study was conducted as part of the eHealth in HD project, which received permission from the ethical committee of the Hospital District of Helsinki and Uusimaa (HUS/1649/2020, Jarkko Ihalainen). All participants gave their voluntary, informed, and written consent. The patients’ capabilities to participate in the study were ensured. While analyzing the data, the information of the patients participating in the study was pseudonymized and coded with the identification numbers. Only the researchers assigned to the study had access to the data. No compensation was paid to the participants for their participation.

### Participants

In total, 18 patients aged ≥60 years participated in the study: 5 patients in predialysis phase, 4 patients in satellite dialysis, 5 patients in home peritoneal dialysis care, and 4 patients in home hemodialysis care (Table 1). Most of the participants were retired. The sample excluded in-center patients with dialysis, but included patients with dialysis in satellite units. The participants were recruited from the group of patients who were participating in the larger research project of HD eHealth solution development.

**Table 1:** Demographics of study participants (N=18).

Demographics			Frequency, n (%)
**Dialysis**			
	Predialysis	5 (28)	
	Satellite dialysis	4 (22)	
	Home peritoneal dialysis	5 (28)	
	Home hemodialysis	4 (22)	
**Gender**			
	Woman	4 (22)	
	Man	14 (78)	
**Education**			
	Basic education	4 (22)	
	Upper secondary level	4 (22)	
	Bachelor’s degree	5 (28)	
	Master’s degree	3 (17)	
	Other	2 (11)	
**Technology skills (self-assessed)**			
	Good	6 (33)	
	Basic	11 (61)	
	Weak	1 (6)	

The health care team recruited the patients by distributing materials about the study and asking about their interest in participation. Participation was voluntary and they did not get any compensation. The study participants were already familiar with the novel HD eHealth solution under development and had tested the first version of the solution. The research nurse contacted the potential participants first and informed them about the study. If the participants wanted to participate in the study, they signed the consent form, and the research nurse provided their contact information to the researchers. Then, the research material package, prepared by the researchers, consisting of a cover letter, a preliminary interview task, background information forms, and the responsible researcher’s contact details were sent to the participants. After sending the package to the participants, the researcher called them to provide more detailed instructions, answer any questions, and schedule a time for the remote interview.

### Preliminary Tasks and Retrospective Interviews

Interviews are useful in the phases of any development process related to eHealth [15]. In our study, retrospective interviews included questions about the patient’s journey, treatments experiences, cooperation with the health care team, technology experiences, and visions for the future. The themes broadly covered patients’ everyday experiences with the illness, practical questions, and the comprehensive timeline with CKD.

The idea of the visual timeline drawing as the preliminary task for the interview was to (1) help the interviewee to process and structure their multistage patient path even before the interview, and (2) help with communication between the interviewee and researcher during the remote interview [23]. The preliminary task was sent to the interviewees 2 weeks before each interview. In this task, patients were asked to identify and illustrate significant milestones, events, and experiences with their illness to a timeline. The patients returned the task to the researchers before the remote interview.

The interviews were conducted between October 2020 and April 2021 when the second wave of COVID-19 was underway in Finland. For safety reasons, the interviews were arranged remotely via Microsoft Teams. Two researchers were present in the interview session with the participating patient: one researcher being the interviewer and the other note-taker. Interviews were audio-recorded using recording functionality of Microsoft Teams. Data gathering was conducted in collaboration with 5 researchers (SL, NK, JV, SH, and PV): 2 doctoral researchers, 2 students, and a professor from the human-computer interaction field.

### Data and Analysis

#### Overview

The data included recordings and notes from the interviews, as well as illustrations of patient journeys. The interview notes were finalized according to audio recordings and the transcriptions were pseudonymized. In total, the data consisted of 155 pages (ie, 58,429 words) of written notes and 18 paper-based patient journey visualizations (ie, visual timeline drawings). A total of 5 researchers (SL, NK, JV, SH, and PV) participated in the data analysis.

**Figure 3:**
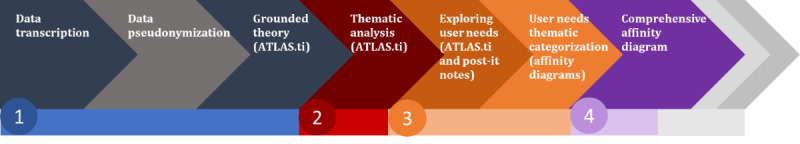
Four phases of data analysis.

The data were analyzed in 4 phases (Figure 3): (1) grounded theory analysis, (2) thematic analysis, (3) user needs exploration and thematic categorization, and (4) comprehensive affinity diagram creation. The data included many different perspectives to understand the different nuances and ensure reliability, and the analysis was done in collaboration with several researchers in many phases.

#### Phase 1: Grounded Theory Analysis

In the first phase [46], the pseudonymized interview transcripts were analyzed following the main structure of grounded theory [47,48] using ATLAS.ti software version 9.1.5.0 (ATLAS.ti Scientific Software Development GmbH, “ATLAS.ti”) [46]. During the analysis, the interview data was coded using researcher-denoted concepts and open coding influenced by the grounded theory [46,47]. The first round of interview data analysis following grounded theory was conducted by SL.

#### Phase 2: Thematic Analysis

In the second phase, the principles of the thematic analysis method [48,49] were followed to analyze the interview data from 18 participants from an existentialist perspective, including references to death, well-being, and atmosphere in life (eg, emotions) [50-52]. In addition, the analysis used the holistic framework [13-15] of exploring the context of use, technology, and people.

On the basis of the analysis, 3 thematic categories were identified: healthy habits, concerns and barriers for eHealth use, and everyday life with CKD. Observations based on interview transcripts were written on post-it notes and thematically categorized following the phases of the affinity diagram method [48,49]. The data gathered using preliminary task of patient journeys were mapped in line with the interview transcripts. In addition to the initial 3 themes, 2 categories were formed based on thematic analysis: digital communication and patients’ emotions. Concerns and use barriers for eHealth were investigated from the data by two researchers (PV and SH). The digital communication affinity diagram was done by three researchers (PV, SH, and JV).

The thematic analysis categories include several aspects as presented in Textbox 1.

**Categories of thematic analysis.**

- Healthy habits: health-promoting activities and hobbies that were mentioned by interviewees, including the whole scale of activities from sports to eating habits.
- Concerns and use barriers for eHealth: observations related to everyday life challenges with the disease, use challenges and barriers of digital services, negative emotions, such as worries or dissatisfactions, and patient path challenges.
- Digital communication: observations concerning health care-related digital communication habits, channels, and experiences.
- Patients’ emotions: observations of emotions and experiences interviewees mentioned in the interviews and patient journey visualizations. The emotions included the whole scale of emotions, from fear and confusion to happiness.
- Everyday life with chronic kidney disease: observations related to interviewees’ thoughts, stories, and experiences of their everyday lives, including references to death. Death made the participants reflect from different perspectives: their own (forthcoming) death was compared to other deaths, the actions to avoid death were listed, and the inevitable nature of death was considered.

Finally, the frequency of observations per theme was calculated (Multimedia Appendix 1). Most of the observations were related to concerns and barriers to technology use. Emotions and healthy habits were also common topics in all the interviews.

#### Phase 3: The User Needs Exploration and Thematic Categorization

The third phase focused on analyzing user needs. All the gathered data were explored from the viewpoint of user needs from 2 perspectives: exploring digital communication needs and exploring overall user needs for interactive solution development. In the analysis, 2 researchers (PV and SH) explored the data labeled “digital communication,” “user needs,” and “to eHealth solution concept” in ATLAS.ti and formulated the observations as user needs. The second round resulted in 165 needs.

After this, the digital communication affinity diagram was created in collaboration with 3 researchers (SH, JV, and PV). The user needs were written on post-it-notes by PV, and analyzed, and regrouped to the affinity diagram in collaboration with SH, JV, and PV. In the affinity diagram, the identified needs (n=165, 100%) were thematically grouped under 5 categories: overall interaction and communication (n=77, 47% of all identified needs), patients’ digital activities (n=28, 17%), instruments (n=27, 16%), inventory and ordering dialysis supplies (n=19, 12%), and digital communication with the health care team (n=14, 8%). While creating the affinity diagram of digital communication needs, doubles were removed.

When exploring overall user needs, the data transcriptions of 18 interviews were analyzed using ATLAS.ti. During this analysis, the data labeled with the following codes—“user needs,” “healthy habits and hobbies,” “suggestion,” “communication,” “digital services,” “tasks,” “care team,” “communication,” “social relationships,” “equipment,” “challenges,” “positive experiences,” and “device research”—were explored and formulated as user needs. In total, 287 user needs were identified and written on post-it notes as preparation for the comprehensive affinity diagram work.

#### Phase 4: Comprehensive Affinity Diagram

In the fourth phase, the affinity diagrams created in the previous analysis phase were merged to form a comprehensive affinity diagram. Three researchers recategorized the post-it notes to create a comprehensive affinity diagram of user needs. The main themes of the diagram were “eHealth user interfaces,” “Inventory and ordering dialysis supplies,” “Overall interaction and communication,” “Process,” “HD eHealth solution,” “Family/peer-support,” “Patients’ digital activities,” and “Needs for improving the quality of life” (Multimedia Appendix 2). These 5 were used as the leading themes to guide the design of the first version of the kidney disease experience map. Data analysis results were discussed between PV, SH, VR, and JV.

### Creation of the First Version of the Kidney Disease Experience Map

**Figure 4:**
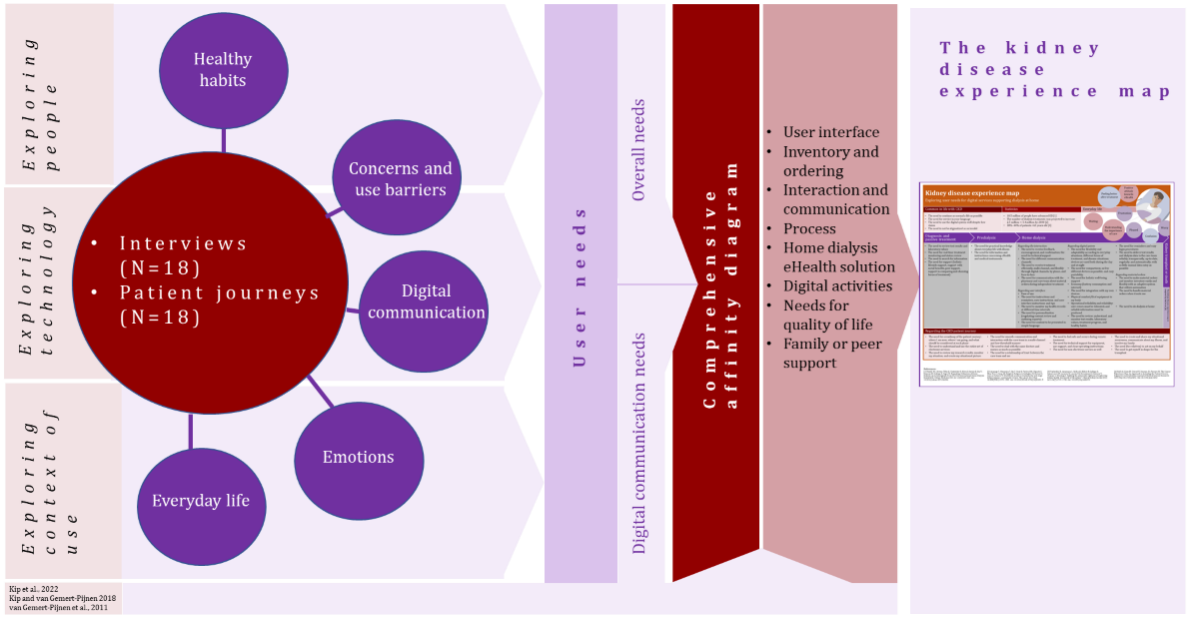
Overview of the data analysis process leading to the creation of the first version of the kidney disease experience map.

An experience map is a tool that strings the perspectives of the context of use, people, and technology, together in a holistic manner [13-15]. The maps can be used to support the communication between stakeholders and to capture a patient’s voice in eHealth design [35]. The first version of the kidney disease patient experience map (Figure 4) was made by combining remarks from existing example models particularly the cancer experience map [35]. In the first paper-based template of our map, the preliminary phases from the preliminary tasks (ie, patient journey maps) were illustrated: “before diagnosis,” “diagnosis and passive treatment,” “treatment method selection,” “beginning of treatment/training,” and “home dialysis.” Post-it notes from the comprehensive affinity diagram were added under those phases. The first draft of the patient experience map was created in collaboration with 3 researchers.

Next, the structure of the experience map was refined based on the number of post-it notes in each phase. The duplicate post-it notes were removed and the content of the map was reorganized (PV, SH, and JV). To validate the contents of the draft map further, 2 researchers (PV and SH) reexamined the post-it notes after which 4 other researchers (JV, NK, SL, and VR) gave feedback on the next version of the map. After all, in total of 6 researchers (PV, SH, JV, NK, SL, and VR) participated at least somehow in the experience map visualization process. The published version of the first version of the kidney disease experience map was finalized by PV. An overview of the multiphase analysis process which led to the creation of this first version of the kidney disease experience map is illustrated in Figure 4.

## Results

### Overview

As the outcome of the study, the first version of the kidney disease experience map, which illustrates the user needs, concerns, and barriers of patients with CKD for eHealth, was created. This section explores the patients (ie, people), technology, and context of use perspectives [13-15]. First, patients’ healthy habits are investigated and their concerns and barriers to technology are addressed. Then, the technology is examined, concentrating on digital communication. Finally, the context of use from the perspective of patients’ everyday lives is explored. Due to sensitive personal issues, research results and citations are presented without identification information to ensure complete anonymity of the participants.

### Exploring the People: Healthy Habits

The patients had different needs for healthy habits regarding social life, sports, and other activities (Multimedia Appendix 3). Patients mentioned that social life and taking care of close relatives, such as grandchildren, were important to them. Incidental exercises, including gardening or shoveling snow, were popular. Being in nature, picking mushrooms or berries, fishing, and hiking, along with other everyday life activities, such as club and society activities or studying, brought well-being to the participants. Animals, for example, dogs, cats, and summer chickens, also supported the participants’ well-being. Handcrafting, art, and culture were also seen as important, but COVID-19 restricted those activities. The importance of many remote social contacts was mentioned in the data, and patients had to learn new eating habits, such as losing weight or cutting down on alcohol consumption.

### Exploring the People: Concerns and Barriers for eHealth Use

Patients were challenged with poorly functioning available eHealth solutions and also experienced other negative factors, such as lack of interest or lack of skills (Multimedia Appendix 4). Technical devices did not always meet the emotional or physical needs of their users. This caused emotions like frustration, worry, and fear from patients. The patients’ responses covered their available eHealth solutions, not focusing especially on the HD eHealth solution.

The solution or software used by patients for their CKD care was found to function poorly in many ways. For example, the devices as a part of the HD eHealth solution did not work as expected, or they worked differently than they expected:

I have no control over the information. If I measure my blood pressure, I press the BT button and then it should be delivered, but I don’t know if it did. The same goes for the scale, *beep*, and maybe it synchronized.

P18

Alongside poorly functioning devices, in some cases, the HD eHealth app also did not work well. The software content (eg, one’s results or visualizations of one’s health situations) was challenging to understand or find. The HD eHealth app was challenging to use with small mobile phone screens and its user interface terminology caused problems for patients:

There have been a few problems with the phone. There will be updates and then it will freeze somehow. I don’t understand much; the basic things are in Finnish, but then they are English words so... This was at least the third time that the nurse had visited us.

P17

Others have also worked well. Even the mobile phone. You can monitor your health from it, even though it has those [health status] curves that you don’t understand much about.

P13

The participants doubted the functionality of the HD eHealth solution, both devices and the app. With the dialysis monitor, they were worried that something would go wrong:

I haven’t used (ie, the devices and instruments) much, I’ve just tried. I’m a bit lazy. I doubt whether the instrument is still in good condition. There was an error message about a month ago. There was a notification that there was still something wrong. I thought I’d let it be and fix the instrument before I started.

P24

When the first treatments started, it was so exciting. But after a month had passed, it had become routine and was no longer exciting. Then, when I moved home from hospital, I started to get excited again.

P22

### Exploring the Technology: Digital Communication

#### Overview

Patients commented on digital devices and the app from many different aspects in terms of the technical functionality and the emotion they cause. The contents of the solution’s user interface (Figure 1), as well as other user interfaces, were also commented on. Patients linked and communicated with the health care team through digital channels and used technology independently as a part of their HD treatment during the research.

#### Many Groups Communicate With the Patient

Communication within the health care team is important, but also nurses, physicians, and patients share information (Multimedia Appendix 2). To get the supplies and instruments needed for HD care, the patient also needs to be in regular contact with the pharmacy or the health care team. Relatives must not be forgotten either, as patients may want to share information with them:

To be able to get in touch with the on-call nephrologist or the other way around, perhaps you could organize a little more frequent meeting. I think that many patients could have questions for the doctor that the nurses cannot or are not allowed to answer. Nephrologist on-call from the computer.

P24

#### Examples of Current Communication Channels

Depending on the patients’ devices and patients’ capabilities to use them, HD patients use different communication channels for following their health records and CKD-specific operations. They order HD supplies once per month. Some patients have created their digital tools (eg, Excel sheets), which calculate the number of needed supplies automatically. After that, the order will be sent via email to the nurse. Still, Excel sheets or other tools for ordering dialysis supplies are not an option for everybody. Therefore, an easy-to-use solution for ordering supplies and managing inventory is needed for HD patients:

Orders must be sent once a month. I have an Excel sheet that automatically keeps the balances. Then, there is a page where you can automatically see what needs to be ordered. I have made Excel sheets myself. It’s quite a job when there are more than 30 different supplies. Syringes... you must know their numbers. Sometimes, I also check some information. If one item is true, then so are all the others. It’s automatic. I then make a .pdf list from the Excel sheet, one A4, and then it goes [by e-mail to the nurse].

P30

In Finland, patients follow their health records via national or local patient portals (eg, “My Kanta Pages” [53] or “MyChart” [54]). Patients with chronic illness have many laboratory tests taken. They appreciate that all their health records are found in one place, but each record is shown in detail separately, too. Therefore, easy-to-read visualizations of test results and current health situations are needed:

MyChart is as hopeless as My Kanta, both of which break down the lab results separately, even if they want to see them as a whole. Applying for your results is difficult.

P37

I look at the results on ‘My Kanta,’ all diagnoses come there, as well as laboratory test results. From there, I can see them all. The information is readable and remains stored. I think ‘My Kanta’ is really good. There you can also see laboratory visits from a certain period if you want to follow a limited period.

P21

#### Positive Attitude Toward Digital Communication

All patients participating in the study seemed to have a positive attitude toward digital communication tools. For example, paper-based dialysis supply orderings may be forgotten and instead, digital tools can step in and help patients with their everyday lives. Patients felt that digital solutions enabled information-seeking and offered more flexibility in meetings with the health care team and other contacts:

Their version is that they hand out an A4 paper. You should fill it in; it’s difficult manually. The risk is that you forget.

P30

We have internet and e-mail. My wife uses them more; I just read. The wife handles banking and other things remotely. I kind of stopped using the computer when I don’t need to. You could probably handle things digitally. I haven’t come across anything like that. I have had to deal with a lot of paper. I have not been approached digitally. Yes, it would be even easier. You could reach the staff at any time.

P24

### Exploring the Context of Use: Everyday Life and Emotions

#### Overview

The main themes related to everyday life were thinking of death, the importance of care, changes in well-being after treatment started, and emotions (Multimedia Appendix 5).

#### The Presence of Death

With kidney disease, death is present in life. It is known that without dialysis or receiving a transplant, one dies. The logic of the disease is unequivocal: when dialysis no longer helps, the person knows they are going to die:

I think that the disease is deadly and must be treated.

P21

Everyone knows that life ends eventually, but I’m not afraid of it in any way.

P1

#### The General Well-Being of the Participants Varied

The patients understood the essentiality of treatment and often felt better after the treatment started. They wished to be able to continue life as normally as possible. One patient (P9) described experiencing leg cramps at night, which had caused them to sleep poorly. After the dialysis treatment started, the cramps stopped. They managed better than before and could even go for walks:

I hope I don’t end up on dialysis. Of course, it is ahead, but hopefully as far as possible. It is a wish.

P20

It’s just that now I feel like I’m getting older. I recently turned 70, so all kinds of unnecessary aches and pains will increase.

P25

#### Everyday Life Is Characterized by Waiting

The health situation of a patient can be improved either with dialysis or a transplant. Having to wait for transplant can have major setbacks; one patient (P24) had to be removed from the transplantation waiting list due to amputation and sepsis, which is why they now focus on keeping themselves in the best possible physical condition through physical activities. Patients were waiting to travel.

#### A Wide Range of Emotions in Everyday Life

A wide range of emotions appeared in the interview data about the patient’s everyday life. Despite the challenges of the disease, most of the patients described their lives with the disease in somewhat positive terms. Everyday life with the disease seemed to go somewhat smoothly.

In addition to positive feelings, patients also expressed negative emotions, such as confusion, fear, irritation, anger, and frustration. Feelings of confusion were caused by changes in health status and events in the patient’s journey, uncertainty of the future steps in the journey, or technology that worked unexpectedly. After receiving the diagnosis, one patient was confused because they did not fully understand what kind of disease the diagnosis was about. Several patients brought up fear in the interviews. Things related to the treatments were especially scary. Most of the patients also described irritation or anger at least sometimes during the patient journey. In addition, half of the patients reported frustration. Health challenges, the difficulty or even failure of a treatment, and the commitment to dialysis treatment were irritating. The most frustrating thing was when HD treatment did not go as well as planned. Another source of frustration was digital systems, equipment, and devices:

And a little fear. I am quite hopeful that I will get there on the transplantation waiting list and that it will be successful at some point. I hope that I can make it there (ie, from a health perspective), and if this continues, then there is nothing to worry about.

P11

Actigraphy, a part of HD eHealth solution, is inconvenient, but there is nothing wrong with the other devices, everything is fine. I have a strong belief that information will go forward. It’s enough for me to see on the scale and blood pressure monitor that my weight and blood pressure are okay.

P18

This home dialysis takes up a lot of space. Do you see these boxes? A huge package comes once a month. The dialysis monitor also takes up quite a lot of space. And then the cabinets, where you put all the treatment consumables. And there is a bottle like this: hand sanitizer comes once a month. Quite a lot of supplies come from the hospital once a month. This room is completely reserved for dialysis.

P30

HD taking up too much space or other resources was both irritating and frustrating. HD requires much space at home, so some patients had to renovate their homes. However, almost half of the patients felt that their living conditions did not require changes due to HD. The patients felt it was important that the dialysis treatment could also be done at the summer cottage.

Positive emotions included joy, optimism, hope, and satisfaction. A third of the patients mentioned joy in the interviews and most patients expressed hope and optimism. They were satisfied with the professional staff and the stability of the current situation. The supportive and successful dialysis made them happy. Keeping their health situation stable was their biggest wish. Patients also mentioned their willingness to continue their hobbies.

The patients were satisfied with both their health condition and the information related to it. Half of them were satisfied with the treatment. The patients were pleased with the eHealth solution in use during the study and the HD treatment was experienced as proceeding smoothly. The nursing staff was considered friendly and knowledgeable:

I have been very satisfied. They, the care team, have guided me, and when I have challenged them, they have accepted challenges and promised to sort things out.

P37

Home dialysis has gone surprisingly well—no major problems.

P34

At least now, this present moment looks bright.

P13

### The First Version of the Kidney Disease Experience Map

**Figure 5:**
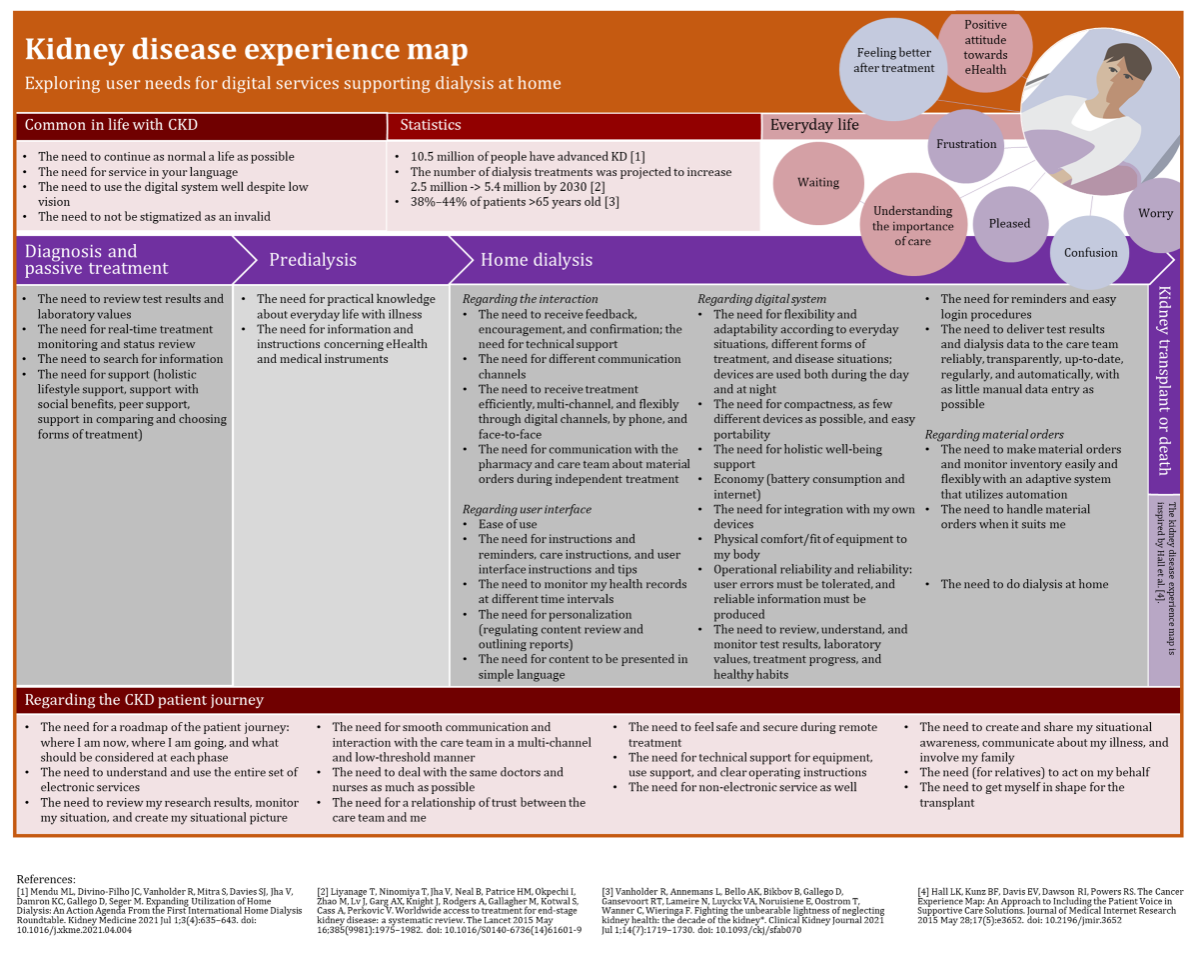
The first version of the kidney disease experience map from a user needs perspective. A higher resolution image is available in Multimedia Appendix 6. CKD: chronic kidney disease. KD: kidney disease.

The first version of the kidney disease experience map (Figure 5 [1-3,35]) was developed from the perspective of user needs of eHealth. It includes their needs for everyday life with CKD (ie, “Common needs”), needs relating to 4 phases of the journey of patients with CKD, and needs in all journey phases of patient with CKD from diagnosis to kidney transplant or death. The patient journey phases, which are inspired by the experience timeline of cancer experience map [35], are “Diagnosis and passive treatment,” “Predialysis,” “HD,” and “Preparing for kidney transplant or death.” In the top corner of our map kidney disease statistics and patients’ everyday life experiences with CKD are illustrated.

The first version of the kidney disease experience map (Figure 5) includes aspects from all three perspectives [13-15]:

1. Context of use: the context of use perspective can be found especially in the patient journey phases and the “everyday life with CKD” part of the map.
2. People: information about people is available in the “statistics and everyday life with CKD” parts of the map.
3. Technology: technology aspects are mainly covered in the form of user needs.

## Discussion

### Principal Findings

On the basis of our study, eHealth as a part of the daily lives of the patients with CKD was perceived as being useful, but the patients also had challenges with devices and apps. Acknowledging that CKD is a long-lasting journey, it is essential to consider long-term user experiences. Finally, several user needs in different phases of the journey of patients with CKD were identified and illustrated with the first version of the kidney disease experience map.

### Answers to Research Questions

#### Overview

This study had three research questions: (1) what experiences do patients, particularly older adults, have in their everyday lives with CKD; (2) what user needs do patients with CKD have for HD eHealth; and (3) how can these needs be illustrated using the experience map technique?

#### What Experiences Do Patients, Particularly Older Adults, Have in Their Lives With CKD?

Three key experiences related to daily life of patients with CKD were identified as depicted in Textbox 2

**Key experiences related to daily life of patients with chronic kidney disease (CKD).**

- Patients’ healthy habits: patients with CKD must adapt to the changes in their everyday lives resulting from dialysis care. For example, patients must learn new routines for their treatment at home. Patients felt that CKD limited their lives, but they dealt with it in different ways: some were frustrated and others continued to live as normally as possible. However, they understood the necessity of the treatment and continued the treatment as usual. Similar findings were reported from a study involving cancer patients [35], who expressed concerns with unknown, life-threatening, and life-changing experiences.
- Patients’ concerns and barriers to use for eHealth: patients had challenges with user interface and technical devices, including dialysis monitors and research devices of HD eHealth solution (Figure 1), that did not always meet the users’ needs.
- Digital communication: digital communication opportunities via the eHealth solution play an important role for patients. Digital solutions made information-seeking possible and offered more flexibility when meeting the health care team and other contacts. Information gaps did not accumulate as easily as for cancer [24].

#### What User Needs Do Patients With CKD Have for HD eHealth?

In our study, both general needs and needs related to different patient journey phases were identified and illustrated in the first version of the kidney disease experience map (Figure 5). We found that CKD was different from day to day, and thus the use context of eHealth varied. Poor usability burdens the everyday life of a person with disease even more. Similar observations were reported in the case of digital independent living supporting systems of older adults [34]: designers were important to understand the treatment stage, the transition from health to illness, and statuses between them. Furthermore, the needs and experiences of patients with cancer were found to vary during the care process [35].

The HD eHealth solution is targeted to all patients with CKD, as well as individuals with disabilities, such as low vision or even amputated limbs. The solution is to be used during the entire patient journey, which may last for years. Considering this, the HD eHealth solution is observed to play a remarkable role in the patient’s life and highlights the need for research on long-term user experience.

#### How Can the User Needs Be Illustrated Using the Experience Map Technique?

Typically, user needs are illustrated with use cases or user needs tables, and they can be processed further to requirements format [55]. Findings from user research can also be presented as user profile illustrations, such as personas [15,34], stakeholder maps [15], experience maps [34], and visualizations of stakeholders [34].

In our study, we used the experience map method to illustrate user needs. As with the cancer experience map [35], we identified the phases of care and relevant themes for grouping the findings. We considered the experience map technique applicable for illustrating not only the phases but also the eHealth related needs of the patients. We found a group of general needs, which are common for all phases of care, but also needs that arise from specific phases of care. Understanding the care process and how user needs evolve is important for the designers and also for the health care team, who are responsible for introducing the services to patients and training them. Experience maps help to ensure the care continuum in service development [34].

In this study, the created experience map represents a common higher-level experience map, which can illustrate a specific persona, or the timeline could be narrower (ie, 1 month or 1 day). In the first version of the kidney disease experience map, the HD eHealth solution covers different wearable solutions and the software (ie, including the wearable solutions and software connected to the dialysis monitors); however, different physical dimensions (ie, dialysis monitors, packing materials of supplies ordered) are not covered.

Experience mapping can be seen as a promising but laborious method for illustrating user needs and communicating a patient’s voice holistically to eHealth development, especially in a case where the experience map is based on extensive qualitative data. Following the example of cancer experience map [35], we captured patient’s voices with their quotes. Similarly, we also focused on reporting patient quotes in the paper instead of the experience map. Therefore, the first version of the kidney disease experience map can be used both separately and with the paper. Our interpretation is that similar to the integrated patient journey map for hypertensive disorders in pregnancy [30] and the cancer experience map [35], the kidney disease experience map can help developers build effective eHealth solutions and promote empathy and shared understanding between the developing team members. It can also help empower patients and help with shared decision-making [33]. Like the patient journey map [24], the first version of the kidney disease experience map has the potential to help simplify and optimize processes for better health outcomes. In addition to that, similar to the cervical dystonia patient journey map [31], it can help to communicate the different phases of the care to the patients and help them to understand their needs at each phase.

### Relevance of the Research

In the study, the experience map describing the patient’s experience was formed based on the qualitative data from retrospective interviews and preliminary tasks. This first version of the kidney disease experience map can be used as a basis for other experience maps related to kidney diseases in the future. In addition, it can also bring perspective to the experience maps of other chronic diseases.

The presented results can be used to support the further development of HD eHealth. The results have already been used in HD eHealth solution development project in Finland (eg, to inform the redesign of the solution user interfaces).

Experience maps, such as the map created in the study, can help researchers, designers, and developers to empathize with the patients with chronic illnesses, and to understand their needs and use contexts for future eHealth innovations. We see our kidney disease experience map and other similar maps having the potential to help design the solutions from a more holistic perspective compared to currently prevailing practices [13,21], as well as to create a base for the new ecosystems and new ways to offer health care in the future [21]. We continue the work in the ongoing research project “Better Health at Home—Optimized Human-Centered Care of Predialysis and Home Dialysis Patients” (2022 to 2026).

### Evaluation of the Study

Our study had some limitations. First, exploring the context of use was not allowed at patients’ homes due to COVID-19 restrictions. Therefore, we needed to collect data remotely. This was partly problematic because the patient’s home is the main context of using HD eHealth. In contrast, due to the patient journey preliminary task and participants’ open-minded attitude during the interviews, we were able to gather rich data about user needs and the context of eHealth use. In our study, the possible impact of the COVID-19 period was considered.

Our sample size is quite small, however, typical for a qualitative interview study. The representativeness of our sample can be questioned as it is divided from a larger patient group based on age and treatment methods. To find as comprehensive a sample as possible, while ensuring the safety of the participants with their ongoing treatment, the health care team helped researchers in recruiting participants for the research study.

Due to the personal nature of health information and privacy, we could not release the participants’ background information, such as specific ages or treatments in this paper. In addition, the indirect identifiers have been removed from the paper. The relative satisfaction of the patients in their lives despite CKD could have been influenced by various factors, besides the HD eHealth solution. First, the target group in this study included only active patients with relatively good physical conditions for research ethics reasons. Second, many of them were waiting for a kidney transplant, which brought them hope with the disease. Overall, there was a lot of variation in emotions, but deep gloom appeared in the data only rarely.

The first version of the kidney disease experience map was created based on a reasonable amount of qualitative data of the everyday lives on patients aged ≥60 years in Finland. Before participating in this study, the patients already knew that the study covered new eHealth solution testing. Therefore, perspectives from patients whose condition is very weak and those whose attitude is not open to new technology are missing in this study. In contrast, research shows that HD is optimal for those who live in areas with long distances to travel to an in-center dialysis unit [6,7]. In part, this can also be seen in the first version of the kidney disease experience map: the study did not include patient experiences during the kidney transplant phase—meaning that the user needs related to this essential phase of the patient journey are missing from our map. In future, the introduced version of the kidney disease experience map can be updated by including motivational issues for HD.

The study was conducted as an integrated part of the patient’s normal CKD treatment, so the research setup was organized in line with the patients’ typical daily conditions and treatments. The study included participants who started using the HD eHealth solution shortly before the interviews but also those who had already been using the solution for several months. Thus, the study covered both novice users and more experienced users. Patients’ reported technology skills varied from weak to good.

### Further Research

This study provided initial experiences of creating an experience map to illustrate patients’ needs for eHealth. In addition, the patients’ experiences from the HD eHealth solution development can offer important perspectives to other development projects regarding patients with CKD. Further research is needed to gather more experience about the applicability of the method. We aim to continue the research with patients with CKD and HD eHealth designs. We will use the illustrated first version of the kidney disease experience map and the listed user needs in further analysis, which will aim to describe design principles and guidelines for HCD of eHealth for patients using HD and other patients with chronic illnesses.

The outcome of the first version of the kidney disease experience map offers insights to the different phases of the patient journey for patients with kidney disease at the HD care. Due to the diverse experiences of patients in different phases of the care process, different types of kidney diseases could also be studied and visualized with experience maps. In addition, the next versions of the kidney disease experience maps could focus on specific days and months. The next versions of the kidney disease experience maps could also be created from the health care team’s perspective.

The content of the first version of the kidney disease experience map was collected with qualitative research methods, which made the consideration of the many different nuances possible. Due to the qualitative nature of the data collection for the first version of the kidney disease experience map, we did not prioritize the available content, for example, based on the amount of the post-it notes. However, without careful prioritization, there is always a risk that the map is too challenging to use as a part of the practical software development work. Therefore, we will involve the patients, as well as the health care team, designers, and software developers, in prioritizing the content of the map in the future.

The perspectives of patients using in-center dialysis are missing from that data, which was the base of the first version of the kidney disease experience map. However, we believe the creation of experience maps for the eHealth needs of patients using in-center dialysis is important for the future as the dialysis modalities might change depending on the patient’s health and life situation.

Our study pointed out the need for further research on the existentialism perspectives of the patients with chronic illnesses using eHealth. Deeper understanding is required on user needs and the role of eHealth as a part of patients’ everyday lives, even when life is nearing its end [50-52].

### Conclusions

eHealth offers possibilities to support the key needs of patients with chronic diseases, such as CKD; however, digital services need to adapt to a wide variety of situations and user needs because kidney diseases are unique to each patient. Patient experiences vary both between and within the stages of treatment. In addition, the patient’s situation, needs, and capacity to use eHealth vary with everyday challenges and opportunities. Furthermore, with older adult patients, the problems that come with age increase the spectrum of barriers to use eHealth and associated challenges.

eHealth should enable proactive communication between the patient and the health care team. Furthermore, awareness of the status should be formed for both parties in real time, as much as possible. In this way, the patients feel safe, are more motivated in their treatment, and take responsibility for themselves.

The experience map is a promising method for illustrating user needs for eHealth, communicating a patient’s voice and describing the longitudinal patient journey to support eHealth development. Our first version of the kidney disease experience map combines people, context of use, and technology perspectives holistically and illustrates the eHealth needs of patients with CKD for the future.

## Appendix

Multimedia Appendix 1 Theme analysis: themes from the second phase of the analysis. CKD: chronic kidney disease.

Multimedia Appendix 2 Main themes of the comprehensive affinity diagram (n=287). Some post-it notes included content for many themes.

Multimedia Appendix 3 Healthy habits and hobbies, and their frequency (n=105, 18 participants).

Multimedia Appendix 4 Barriers to eHealth use (n=288, 18 participants).

Multimedia Appendix 5 Aspects of everyday life with chronic kidney disease, N=18 (aspects N>3 are excluded from the table)

Multimedia Appendix 6 The first version of the kidney disease experience map from a user needs perspective. KD: kidney disease. CKD: chronic kidney disease.

## Abbreviations

CKD: chronic kidney disease
HCD: human-centered design
HD: home dialysis

## Data Availability

The datasets generated or analyzed during this study are not publicly available due to the ethical reasons as stated in the research permit application.
