# Supplementary material for: Illustrating User Needs for eHealth With Experience Map: Interview Study With Chronic Kidney Disease Patients
Source: JMIR Hum Factors. 2025 Mar 18;12:e48221. doi: 10.2196/48221 (PMC11962329; doi:10.2196/48221)
Supplement: Multimedia Appendix 1 [file humanfactors_v12i1e48221_app1.pdf]

| Theme | Frequency / mentions (N) | Frequency / Participants (N/18, %) |
|---|---|---|
| | | |
| Healthy habits | 106 | 18/18, (100) |
| Concerns and use barriers of eHealth | 299 | 18/18, (100) |
| Digital communication | 29 | 14/18, (78) |
| Expressions and patients' emotions | 293 | 18/18, (100) |
| Everyday life with CKD (Death) | 15 | 13/18, (72) |
