# Supplementary material for: Illustrating User Needs for eHealth With Experience Map: Interview Study With Chronic Kidney Disease Patients
Source: JMIR Hum Factors. 2025 Mar 18;12:e48221. doi: 10.2196/48221 (PMC11962329; doi:10.2196/48221)
Supplement: Multimedia Appendix 2 [file humanfactors_v12i1e48221_app2.pdf]

Theme	Number of tags (=post-it notes) (%)
eHealth user interfaces (content and functions)	26 (9)
Inventory and ordering dialysis supplies	19 (6)
**Overall interaction and communication**	Total 77 (27)
A. Need for information in different phases of the patient's path	A. 15 (5)
B. Need for guidance and support	B. 11 (4)
C. Data transfer	C. 11 (4)
D. Digital communication with care team	D. 14 (5)
E. Feedback on devices of eHealth solution	E. 11 (4)
F. Need for care team	F. 13 (5)
Process	33 (11)
Home dialysis eHealth solution	27 (9)
Family and peer-support	13 (5)
Patients' digital activities	28 (10)
Needs for improving the quality of life	14 (5)
