# Supplementary material for: Illustrating User Needs for eHealth With Experience Map: Interview Study With Chronic Kidney Disease Patients
Source: JMIR Hum Factors. 2025 Mar 18;12:e48221. doi: 10.2196/48221 (PMC11962329; doi:10.2196/48221)
Supplement: Multimedia Appendix 3 [file humanfactors_v12i1e48221_app3.pdf]

Healthy habit themes	Frequency / mentions N (%)	Frequency / Participants N (%)
Social life and taking care of close relatives	17 (16)	11 (61)
Nature (picking mushrooms and berries, fishing, hiking)	15 (14)	11 (61)
Everyday life activities, such as club and society activities, studying	15 (14)	11 (61)
Incidental activities (such as gardening or shoveling snow)	12 (11)	6 (33)
Traveling	6 (6)	6 (33)
Physical activities such as swimming, walking, or jogging	6 (6)	6 (33)
Handicrafts, art, and culture	6 (6)	4 (22)
Social contacts	5 (5)	4 (22)
Summer cottage	4 (4)	4 (22)
Previously important activities can now be impossible	4 (4)	4 (22)
Pets (dogs, cats)	4 (4)	3 (17)
Other	11 (10)	-
