# Supplementary material for: Illustrating User Needs for eHealth With Experience Map: Interview Study With Chronic Kidney Disease Patients
Source: JMIR Hum Factors. 2025 Mar 18;12:e48221. doi: 10.2196/48221 (PMC11962329; doi:10.2196/48221)
Supplement: Multimedia Appendix 4 [file humanfactors_v12i1e48221_app4.pdf]

Theme	Frequency, mentions N (%)	Frequency, participants N (%)	Subthemes
Poorly functioning system or software	58 (20)	18 (100)	Inoperative device<br>Inoperative software<br>National patient portal's website is confusing, hard for patients to understand their own medical results.
Experienced challenges with technologies	54 (19)	14 (78)	Worry of bothering care team<br>Fear that something will go wrong with eHealth<br>Lack of instructions, for example, when errors occur<br>Dissatisfaction<br>Lack of overall picture of one's own illness<br>Frustration
Other barriers for digital service use	7 (2)	7 (39)	Continuous follow-up of different results may cause stress<br>Does not match the mental model: did not find the necessary information from the eHealth solution (Figure 1)
Lack of interest	4 (1)	4 (22)	No need for peer support<br>Notifications about inoperability reduce motivation
Lack of capacity	3 (1)	2 (11)	Problems with eyesight -> hard to use the interface<br>Screen of mobile phone is not large enough, which makes scrolling large amounts of content difficult.
Lack of competence	8 (3)	4 (22)	Digital services (digital questionnaire) are hard to find. The service address is too complicated. The log-in is difficult as it requires a strong identification requirement.<br>Wrong operating system (e.g., Android, IOS)<br>Challenges with devices<br>Challenges to understand the services' contents
Expressions for challenges with technology	A: 34 (12)<br>B: 69 (24)	18 (100)<br>A: 11 (61)<br>B: 14 (78)	A: Worry or fear: Worry of bothering care team; Fear that something goes wrong with technology; Lack of instructions, for example, when errors occur; Dissatisfaction; Lack of overall picture of own illness<br>B: Frustration: Some specific phase of the patient journey, challenges with care team and other people, challenges with devices
