# Supplementary material for: Illustrating User Needs for eHealth With Experience Map: Interview Study With Chronic Kidney Disease Patients
Source: JMIR Hum Factors. 2025 Mar 18;12:e48221. doi: 10.2196/48221 (PMC11962329; doi:10.2196/48221)
Supplement: Multimedia Appendix 5 [file humanfactors_v12i1e48221_app5.pdf]

Theme	Frequency, mentions N (%)	Frequency, participants N (%)	Sub-themes
Thinking of death	16 (100)	12 (67)	Without dialysis, patient dies; when dialysis no longer helps<br>Dying at some point
**Current overall situation and Importance of care**	28 (100)	16 (89)	
	A: 6 (21)	A: 3 (17)	A: Moving dialysis phase forward, but soon dialysis treatment will start
	B: 5 (18)	B: 4 (22)	B: The condition improves after the treatment has started
	C: 8 (29)	C: 6 (33)	C: I feel well
	D: 7 (25)	D: 6 (33)	D: I have health challenges
	E: 6 (21)	E: 6 (33)	E: Everyday life works well
Change in wellbeing	9 (100)	7 (39)	Changes with health status and patient journey
**Waiting**	29 (100)	13 (72)	CKD care is full of waiting
	A: 19 (66)	A: 10 (56)	A: kidney transplant
	B: 3 (10)	B: 3 (17)	B: death
	C: 5 (17)	C: 5 (28)	C: to travel again
**Emotions**	276 (100)	18 (100)	
	A(F): 28 (10)	A(F): 11 (61)	A(F): Irritation (as a feeling)
	af1: 7 (3)	af1: 5 (28)	af1: The difficulty of a treatment or the failure of a treatment
	af2: 4 (1)	af2: 4 (22)	af2: Commitment to dialysis
	af3: 3 (1)	af3: 3 (17)	af3: Letting go of one's important hobbies
	af4: 4 (1)	af4: 3 (17)	af4: Difficult operation or ergonomics of the system, equipment, or instrument
	af5: 3 (1)	af5: 3 (17)	af5: Change of care team
	A (E): 69 (25)	A (E): 14 (78)	A(E): Irritation as an expression for challenges with technology
	B: 15 (5)	B: 8 (44)	B: Confusion
	b1: 7 (3)	b1: 6 (33)	b1: Changes in health and patient journey
	C: 23 (8)	C: 10 (56)	C: Fear

	c1: 4 (1)	c1: 3 (17)	c1: Matters related to treatments
	c2: 4 (1)	c2: 3 (17)	c2: Weakening of health
	D: 12 (4)	D: 6 (33)	D: Anger
	d1: 3 (1)	d1: 3 (17)	d1: A certain stage of the patient journey, such as the challenges of getting a diagnosis
	E: 13 (5)	E: 10 (56)	E: Frustration
	e1: 3 (1)	e1: 3 (17)	e1: The HD treatment was not as successful as hoped
	G: 115 (42)	G: 18 (100)	G: Contentment
	g1: 26 (9)	g1: 12 (67)	g1: Information on health status and treatment
	g2: 35 (13)	g2: 15 (83)	g2: Friendly and competent care team
	g3: 10 (4)	g3: 8 (44)	g3: Satisfied with life in general (wellbeing has been good and wellbeing has been good with the help of home dialysis)
	g4: 3 (17)	g4: 3 (17)	g4: Flexible, easy care
	H: 22 (8)	H: 13 (72)	H: Hope
	h1: 16 (6)	h1: 8 (44)	h1: Hope for a kidney transplant
	h2: 5 (2)	h2: 5 (28)	h2: Hope the situation remains stable
	I: 6 (2)	I: 6 (33)	I: Delight (Competent care team; The stability of the situation and a good current situation)
**Living conditions and home dialysis**	54 (100)	18 (100)	
	A: 6 (11)	A: 4 (22)	A: Need to shake-up home due to home dialysis
	B: 10 (19)	B: 8 (44)	B: No need to shake-up home due to home dialysis
	C: 5 (9)	C: 4 (22)	C: Dialysis must be able to be done from summer cottage
	D: 6 (11)	D: 5 (28)	D: HD takes a lot of space at home
Pain	10 (100)	8 (44)	Disease-related pain, Age-related pain, No pain
