# Supplementary figures and images for: Illustrating User Needs for eHealth With Experience Map: Interview Study With Chronic Kidney Disease Patients
Source: JMIR Hum Factors. 2025 Mar 18;12:e48221. doi: 10.2196/48221 (PMC11962329; doi:10.2196/48221)

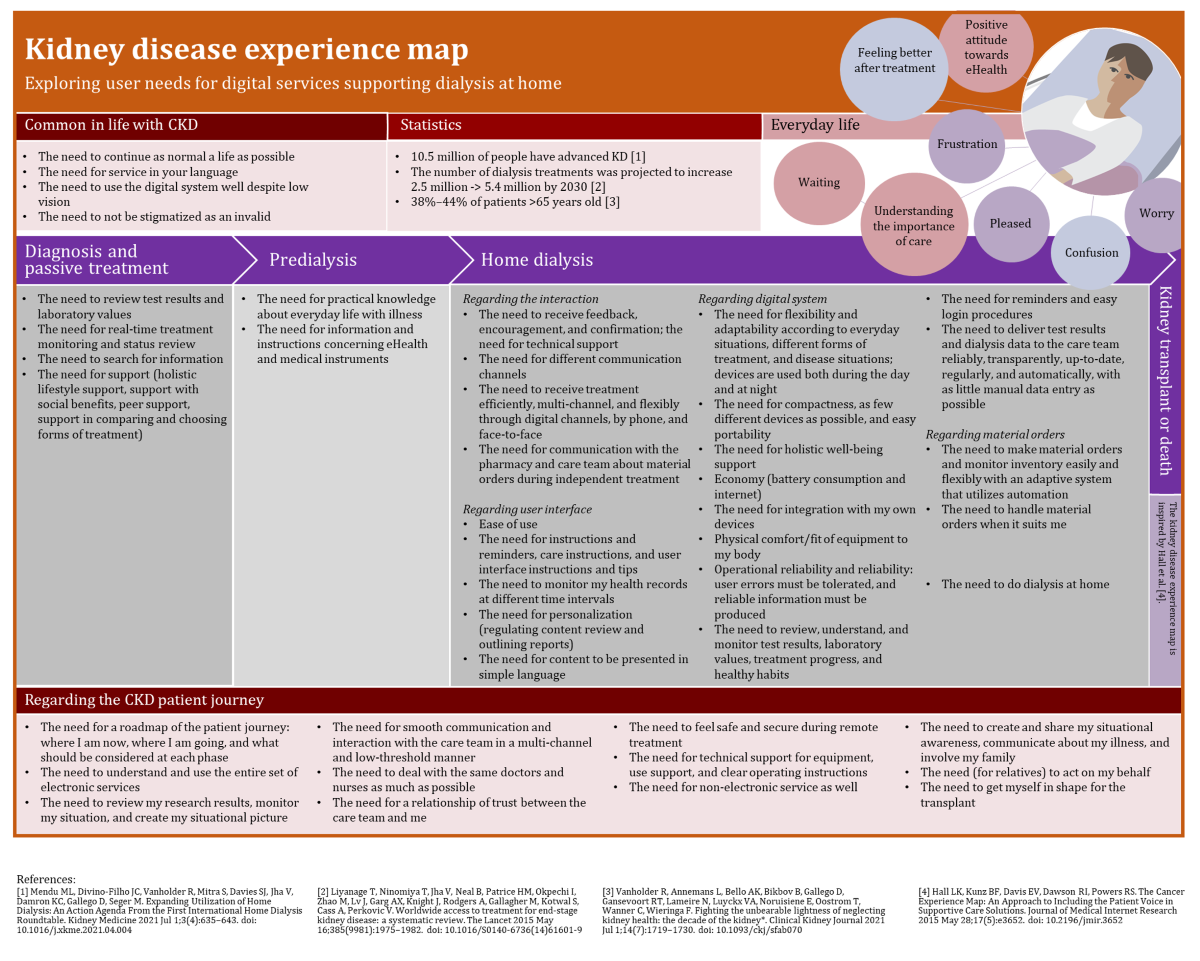

Supplement: Multimedia Appendix 6 [file humanfactors_v12i1e48221_app6.png]
